# Supplementary material for: Comprehensive Analysis of Gene Expression Profiling to Explore Predictive Markers for Eradication Therapy Efficacy against Helicobacter pylori-Negative Gastric MALT Lymphoma
Source: Cancers (Basel). 2023 Feb 14;15(4):1206. doi: 10.3390/cancers15041206 (PMC9954119; doi:10.3390/cancers15041206)
Supplement: Supplementary file 1 [file cancers-15-01206-s001.zip › cancers-2208385-supplementary.pdf]

Supplementary Material

Table S1. Patient backgrounds according to *Helicobacter* pylori infection in gastric MALT lymphoma

		**Hp+ (n = 87)**	**Hp- (n = 50)**	**P-value**
Median age (range)		61.3 ± 12.5 (45–82)	61.5 ± 12.6 (32–88)	0.924
Sex	Male	42 (48%)	18 (36%)	0.211
	Female	45 (52%)	31 (64%)	
Mucosal atrophy	Closed-type	45 (52%)	44 (88%)	< 0.001*
	Open-type	42 (48%)	6 (12%)	
Effect of eradication therapy	CR	64 (74%)	17 (34%)	< 0.001*
	NC	23 (26%)	33 (66%)	
Number of lesions	Single	33 (38%)	14 (28%)	0.267
	Multiple	54 (62%)	36 (72%)	
Location	U	11 (13%)	12 (24%)	0.0814
	M/L	76 (87%)	38 (76%)	
Morphological type	Superficial	64 (74%)	42 (84%)	0.160†
	Elevated	15 (17%)	4 (8%)	0.131†
	Other	8 (9%)	4 (8%)	0.811†
API2-MALT1 gene	Present	3 (4%)	14 (28%)	< 0.001*
	Absent	81 (96)	36 (72%)	

Fisher’s exact test was performed for categorical variables.

Mann–Whitney *U* test was performed for comparative analyses of continuous variables.

* Statistically significant.

† Significance level ($P < 0.05/3$) was adjusted with Bonferroni correction for multiple comparisons.

U, upper part of stomach; M, middle part of stomach; L, lower part of stomach

API2-MALT1, API2-MALT1 chimeric transcript; Hp, Helicobacter pylori; CR, complete response (ChR and pMRD according to GELA histological grading system); NC, no change (rRD and NC according to GELA histological grading system)

Table S2. Primers for validation study

Target gene	Direction	Sequence (5′-3′)	Product size (bp)	References
h-GAPDH	Forward	TCCCTGAGCTGAACGGGAAG	217	[55]
	Reverse	GGAGGAGTGGGTGTCGCTGT		
ALAS2	Forward	CGGGGCGCTGGGATTGG	173	[56]
	Reverse	GGGGGCAGAGAAGTGGTAAAGATG		
OLFM4	Forward	ACTGTCCGAATTGACATCATGG	135	[57]
	Reverse	TTCTGAGCTTCCACCAAAACTC		
HBA1	Forward	GGTCCCCACAGACTCAGAGA	287	[58]
	Reverse	AGTGCGGGAAGTAGGTCTTG		

HBA2	Forward	CTGGACAAGTTCCTGGCTTC	165	[58]
	Reverse	TGCTGCCCACTCAGACTTTA		
HBB	Forward	GCAACCTCAAACAGACACCA	313	[59]
	Reverse	CAGCATCAGGAGTGGACAGA		
HLA-DRB3	Forward	CATGGTGTGTCTGAAGCTCCC	140	Self-designed, ACCESSION NM_022555
	Reverse	AGAAATGACACTCAGACTTACGCAG		
NPIPB3	Forward	TCGTGGGACTGACTTTGGTG	215	Self-designed, NM_130464.3 *Homo sapiens* nuclear pore complex interacting protein family member B3 (NPIPB3), mRNA
	Reverse	AAATGGACCTCAGCCCTTCC		
LEP	Forward	GAAGACCACATCCACACACG	189	[60]
	Reverse	AGCTCAGCCAGACCCATCTA		
MTRNR2L8	Forward	TGCCCAGTGACATGCGTTT	235	Self-designed, NM_001190702.2 *Homo sapiens* MT-RNR2 like 8 (MTRNR2L8), mRNA
	Reverse	GGCCTGTGGACTTGTTAAGTG		
NANOG	Forward	CCCAAAGGCAAACAACCCACTTCT	107	[61]
	Reverse	AGCTGGGTGGAAGAGAACACAGTT		
OTUD6A	Forward	TGGATGATCCGAAGAGTGAAC	202	[62]
	Reverse	TCTTGGAACTTCTCCAGCTCCT		

ZNF669	Forward CGCTGCTAGTTCTTGGCTCT<br>Reverse CACTCACCATTCCTCGGCTT	128	Self-designed, NM_024804.3 (transcript variant [55]) & NM_001142572.2 (transcript variant [56])
TMEM130	Forward CCAGCCATTCACCCATCTGT<br>Reverse CTGGGAGTCAACACGGAACA	133	Self-designed, BC037895
GSTA1	Forward CAGCAAGTGCCAATGGTTGA<br>Reverse TATTTGCTGGCAATGTAGTTGAGAA	80	[58]
ZNF556	Forward AGTGTGGGAAAGCCTACTGC<br>Reverse TTTGACATCTGTGGAGGGGC	215	[58]
ACTA1	Forward CTAGACACACTCCACCTCCA<br>Reverse GTCAGTTTACGATGGCAGCA	132	[59]
APOBEC3A	Forward GAGAAGGGACAAGCACATGG<br>Reverse TGGATCCATCAAGTGTCTGG	61	[63]

Supplementary Methods

Diagnosis and staging of gastric MALT lymphoma

Gastric MALT lymphoma was diagnosed according to the criteria of the World Health Organization [64] and if the symptoms were consistent with grade 4 or 5 of the Wotherspoon

histological scoring system [65]. Our study included patients with atypical lymphocytes that were positive for CD20 and CD79a and negative for CD3. After eradication therapy, histopathological evaluation was performed using the Group d'Etude des Lymphomes de l'Adulte (GELA) grading system [30]. CR of lymphoma was defined as complete histological response (ChR) or probable minimal residual disease (pMRD) in the Ann Arbor staging system [67]. Tumor stage was evaluated via clinical examination; full blood count; biochemical analysis; and cervical, pectoral, abdominal, and pelvic computed tomography scans.

Eradication therapy

To eradicate *Helicobacter* species, patients with gastric MALT lymphoma received a 1-week course of oral PPI (lansoprazole 30 mg)/P-CAB (Vonoprazan fumarate 20mg), amoxicillin 750 mg, and clarithromycin (200/400 mg) twice a day as first-line eradication therapy. The choice of PPI/P-CAB and dose of clarithromycin were based on the patients' condition and time period. Here, treatment with this regimen was defined as "eradication therapy," regardless of Hp infection status. In Hp-positive patients, when the Hp was not successfully eradicated following first-line eradication therapy, a second-line eradication therapy regimen (PPI/P-CAB, amoxicillin 750 mg, and metronidazole 250 mg, twice a day) was administered. Conversely, in Hp-negative gastric MALT lymphoma cases, eradication therapy was conducted only once with first-line eradication

therapy regimen.

RNA extraction and reverse transcription

Total RNA was extracted from biopsy specimens of gastric MALT lymphoma tumor lesions before therapeutic intervention (eradication therapy) using an RNeasy Kit (Qiagen, Tokyo, Japan). cDNA was synthesized from 1 μg total RNA using a first-strand cDNA synthesis kit (Amersham Biosciences, Piscataway, NJ, USA). Reverse transcription of RNA into cDNA was performed with a first-strand cDNA synthesis kit (Amersham Biosciences, Buckinghamshire, UK) [68].

Quantitative reverse transcription polymerase chain reaction

Quantitative reverse transcription polymerase chain reaction (qRT-PCR) was performed using a LightCycler FastStart DNA Master SYBR Green I Kit (Roche Diagnostics, Basel, Switzerland). To correct for differences in sRNA quality and quantity between samples, expression values were reported, normalized to glyceraldehyde-3-phosphate dehydrogenase (*GAPDH*), and subsequently mean-centered. Primers for PCR were designed as previously described [69], and other primer sequences were determined according to previous reports [55-63]. Primer sequences are listed in Table S2.

Transcription factor prediction of DEGs

We used the getorf program to find open reading frames (ORFs) of each DEG. For plants, we aligned ORFs to transcription factor (TF) domains (from PlntfDB) using hmmsearch [70]; for animals, we aligned ORFs to the animal TF database (AnimalTFDB) using DIAMOND [71].

Software information: PlntfDB, Version: v23.0. (http://plntfdb.bio.uni-potsdam.de/v3.0/)

AnimalTFDB: Version: v2.0. Website: http://www.bioguo.org/AnimalTFDB/

getorf: Version: EMBOSS:6.5.7.0 Parameters: -minsize 150 (http://www.bioinformatics.nl/cgi-bin/emboss/help/getorf)

hmmsearch: Version: v3.0. Parameters: default. (http://hmmer.org)

DIAMOND: Version: v0.8.31. Parameters: --more-sensitive --evalue 1e-5. (https://github.com/bbuchfink/diamond)

PPI analysis of DEGs

We used DIAMOND [71] to map the DEGs to the STRING [39] database to obtain the interaction between DEG-encoded proteins using homology with known proteins. We selected the top 100 interaction networks to draw the picture; for the entire interaction result, we used an input file that could be imported directly into Cytoscape for network analysis. Cytoscape is a software for complex network analysis and visualization (https://cytoscape.org/).

STRING: Version: v10, Website: http://string-db.org/

DIAMOND: Version: v0.8.31, Parameters (Running): --evalue 1e-5 --outfmt 6 --max-target-seqs 1 --more-sensitive. Parameters (Selecting): query coverage ≥ 50%, identity ≥ 40% (https://github.com/bbuchfink/diamond)

Supplementary References

55. Zainuddin, A.; Chua, K. H.; Abdul Rahim, N.; Makpol, S. Effect of Experimental Treatment on GAPDH mRNA Expression as a Housekeeping Gene in Human Diploid Fibroblasts. *BMC Mol. Biol.* **2010**, *11*, 59.
56. Kaneko, K.; Furuyama, K.; Aburatani, H.; Shibahara, S. Hypoxia Induces Erythroid-Specific 5-Aminolevulinate Synthase Expression in Human Erythroid Cells Through Transforming Growth Factor-Beta Signaling. *FEBS J.* **2009**, *276*, 1370–1382.
57. Ma, H.; Tian, T.; Liang, S.; Liu, X.; Shen, H.; Xia, M.; Liu, X.; Zhang, W.; Wang, L.; Chen, S.; Yu, L. Estrogen Receptor-Mediated miR-486-5p Regulation of OLFM4 Expression in Ovarian Cancer. *Oncotarget* **2016**, *7*, 10594–10605.
58. Vishnubalaji, R.; Sasidharan Nair, V.; Ouararhni, K.; Elkord, E.; Alajez, N. M. Integrated Transcriptome and Pathway Analyses Revealed Multiple Activated Pathways in Breast Cancer. *Front. Oncol.* **2019**, *9*, 910.
59. Tian, Z.; Palmer, N.; Schmid, P.; Yao, H.; Galdzicki, M.; Berger, B.; Wu, E.; Kohane, I. S. A Practical Platform for Blood Biomarker Study by Using Global Gene Expression Profiling of Peripheral Whole Blood. *PLOS ONE* **2009**, *4*, e5157.
60. Kumar, J.; Fang, H.; McCulloch, D. R.; Crowley, T.; Ward, A. C. Leptin Receptor Signaling via Janus Kinase 2/Signal Transducer And Activator Of Transcription 3 Impacts on Ovarian Cancer Cell Phenotypes. *Oncotarget* **2017**, *8*, 93530–93540.
61. Park, S. W.; Do, H. J.; Choi, W.; Song, H.; Chung, H. J.; Kim, J. H. NANOG Gene Expression Is Regulated by the ETS Transcription Factor ETV4 in Human Embryonic Carcinoma NCCIT Cells. *Biochem. Biophys. Res. Commun.* **2017**, *487*, 532–538.
62. Kim, S. Y.; Kwon, S. K.; Lee, S. Y.; Baek, K. H. Ubiquitin-Specific Peptidase 5 and Ovarian Tumor Deubiquitinase 6A Are Differentially Expressed in p53+/+ and p53−/− HCT116 Cells. *Int. J. Oncol.* **2018**, *52*, 1705–1714.
63. Burns, M. B.; Lackey, L.; Carpenter, M. A.; Rathore, A.; Land, A. M.; Leonard, B.; Refsland, E. W.; Kotandeniya, D.; Tretyakova, N.; Nikas, J. B.; Yee, D.; Temiz, N. A.; Donohue, D. E.; McDougle, R. M.; Brown, W. L.; Law, E. K.; Harris, R. S. APOBEC3B Is an Enzymatic Source of Mutation in Breast Cancer. *Nature* **2013**, *494*, 366–370.
64. Swerdlow, S. H.; Campo, E.; Pileri, S. A.; Harris, N. L.; Stein, H.; Siebert, R.; Advani, R.; Ghielmini, M.; Salles, G. A.; Zelenetz, A. D.; Jaffe, E. S. The 2016 Revision of the World Health Organization Classification of Lymphoid Neoplasms. *Blood* **2016**, *127*, 2375–2390.
65. Wotherspoon, A. C.; Ortiz-Hidalgo, C.; Falzon, M. R.; Isaacson, P. G. Helicobacter pylori-Associated Gastritis and Primary B-Cell Gastric Lymphoma. *Lancet* **1991**, *338*, 1175–1176.
66. Copie-Bergman, C.; Gaulard, P.; Lavergne-Slove, A.; Brousse, N.; Fléjou, J. F.; Dordonne, K.; de Mascarel, A.; Wotherspoon, A. C. Proposal for a New Histological Grading System for Post-treatment Evaluation of Gastric MALT Lymphoma. *Gut* **2003**, *52*, 1656.
67. Musshoff, K. [Clinical Staging Classification of Non-Hodgkin's Lymphomas (Author's Transl)]. *Strahlentherapie* **1977**, *153*, 218–221.

68. Yuge, R.; Kitadai, Y.; Takigawa, H.; Naito, T.; Oue, N.; Yasui, W.; Tanaka, S.; Chayama, K. Silencing of Discoidin Domain Receptor-1 (DDR1) Concurrently Inhibits Multiple Steps of Metastasis Cascade in Gastric Cancer. *Transl. Oncol.* **2018**, *11*, 575–584.
69. Takigawa, H.; Kitadai, Y.; Shinagawa, K.; Yuge, R.; Higashi, Y.; Tanaka, S.; Yasui, W.; Chayama, K. Mesenchymal Stem Cells Induce Epithelial to Mesenchymal Transition in Colon Cancer Cells Through Direct Cell-to-Cell Contact. *Neoplasia* **2017**, *19*, 429–438.
70. Mistry, J.; Finn, R. D.; Eddy, S. R.; Bateman, A.; Punta, M. Challenges in Homology Search: HMMER3 and Convergent Evolution of Coiled-Coil Regions. *Nucleic Acids Res.* **2013**, *41*, e121
71. Buchfink, B.; Xie, C.; Huson, D. H. Fast and Sensitive Protein Alignment Using DIAMOND. *Nat. Methods* **2015**, *12*, 59–60.
